# The Mixed Addition of Biochar and Nitrogen Improves Soil Properties and Microbial Structure of Moderate–Severe Degraded Alpine Grassland in Qinghai-Tibet Plateau

**DOI:** 10.3389/fpls.2021.765041

**Published:** 2021-11-22

**Authors:** Jinsheng Li, Yinquan Zhao, Xinqing Shao, Ding Huang, Jianying Shang, Hui Li, Yixuan He, Kesi Liu

**Affiliations:** ^1^College of Grassland Science and Technology, China Agricultural University, Beijing, China; ^2^College of Tourism and Urban Planning, Chengdu University of Technology, Chengdu, China; ^3^College of Land Science and Technology, China Agricultural University, Beijing, China; ^4^Key Laboratory of Restoration Ecology of Cold Area in Qinghai Province, Northwest Institute of Plateau Biology, Chinese Academy of Sciences, Xining, China; ^5^National Field Station of Grassland Ecosystem, Guyuan, China

**Keywords:** alpine grassland, degradation, biochar, soil physicochemical property, microbial community

## Abstract

The degradation of the grassland system has severely threatened the safety of the ecological environment and animal husbandry. The supplement of key substances lost due to degradation is widely used to accelerate the restoration of the degraded grassland ecosystem. In this study, we investigated the effects of biochar and nitrogen addition on soil properties and microorganisms of degraded alpine grassland. The experimental treatments consisted of the control without any addition, only nitrogen addition (10 gN/m^2^), only biochar addition (4.00 kg/m^2^ biochar), and the mixed addition of biochar and nitrogen (4.00 kg/m^2^ biochar and 10 gN/m^2^ nitrogen, respectively). Adding N alone did not significantly change the pH, total organic carbon (TOC), total nitrogen (TN), microbial biomass (MB), and the composition proportion of microbes of the soil, but increased the contents of soil water content (SWC), NH_4_^+^-N, NO_3_^–^-N, available phosphorus (AP), and the biomass of bacteria and fungi. The addition of biochar or the mixture of biochar and nitrogen increased the contents of pH, TOC, TN, MB, SWC, NH_4_^+^-N, NO_3_^–^-N, AP, bacteria, and fungi in the soil and changed the structure of the soil microbial community. The increasing intensity of AP, bacteria, and fungi under the addition of biochar or the mixture of biochar and nitrogen was significantly greater than that under N addition alone. These results indicated that the separated addition of nitrogen and biochar and the mixed addition of biochar and nitrogen all improved the soil condition of the moderate–severe degraded alpine grassland, but the mixed addition of biochar and nitrogen could be a better strategy to remediate the degraded alpine grassland.

## Introduction

The alpine grassland ecosystem in the Qinghai-Tibet Plateau is a unique ecosystem formed in a high-altitude environment, accounting for 50.9% areas of the Qinghai-Tibet Plateau and 44% areas of China grasslands (Piao et al., 2012). As the main ecosystem of the Qinghai-Tibet Plateau, alpine grassland is not only the basis of local animal husbandry but also has a variety of ecological functions, such as water conservation, soil erosion control, biodiversity maintenance, and carbon sequestration (Feng et al., 2009; Li et al., 2014; Sun et al., 2019). However, due to harsh climate, short growth period, weak resistance, and poor resilience, the alpine grassland ecosystem is relatively fragile (Feng et al., 2009; Liu et al., 2018). Due to unreasonable grazing utilization, grasslands on the Qinghai-Tibet Plateau underwent a large area of degradation between the end of the 19th century and the beginning of the 20th century (Yao et al., 2016; Li et al., 2020). The degradation of alpine grassland leads to the decline of grassland vegetation, the deterioration of soil habitat, the decrease of grassland productivity and ecological functions, and finally the serious imbalance of the whole ecosystem (Yao et al., 2016; Peng et al., 2018).

The deterioration of soil habitat caused by grassland degradation is mainly due to the destruction of soil structure and the sharp loss of soil nutrients, especially soil nitrogen and carbon, which destroy the soil biological system and the growth environment of plant roots (Liu et al., 2020; Sun et al., 2020; Wu et al., 2021). For example, a study in the Qinghai-Tibet Plateau found that the nitrogen content in the soil decreased by 49% after the degradation of grassland (Dong et al., 2012). Nitrogen is an essential nutrient for plant growth and microbial development and thus limits the productivity of natural grassland ecosystems (Wang D. et al., 2020). The reduction of soil nitrogen not only restricts the growth of aboveground vegetation (Li et al., 2020) but also reduces the microorganisms in the soil due to the lack of necessary nutrients (Che et al., 2019). In view of the lack of soil nitrogen after grassland degradation, nitrogen fertilizer application is a conventional method to increase soil nutrients. Nitrogen application can increase the content of available nitrogen and total organic carbon (TOC; Luo et al., 2011), improve the utilization efficiency of nitrogen and carbon by microorganisms (Li et al., 2018), and increase the productivity of the ecosystem (Somleva et al., 2002). In the Qinghai-Tibet Plateau, previous studies found that adding nitrogen not only changes the composition of soil nitrogen but also has a positive effect on aboveground vegetation (Fu and Shen, 2016, 2017). However, in the degraded grassland, the soil generally has high bulk density, low porosity, low organic matter, and unstable aggregate structure (Ma et al., 2020; Zhang et al., 2020). These traits make soil difficult to retain the introduced nutrients and result in the loss of nutrients and the decrease of utilization efficiency (Yagüe et al., 2016; Zhang et al., 2018).

Biochar is a highly aromatic and carbon-rich substance formed by high-temperature pyrolysis and carbonization of biomass under oxygen-limited or anaerobic conditions (Woolf et al., 2010). As biochar has porous, large specific surface area, strong adsorption capacity, rich carbon content, and other properties, it is widely used in agricultural land as an excellent soil improvement material (Butnan et al., 2017; El-Naggar et al., 2018; Muhammad et al., 2018). The bulk density of biochar is around 0.08–0.5 g/cm^3^, smaller than that of soil (1.0–1.7 g/cm^3^), and therefore, the biochar application significantly decreases the bulk density of the soil (Wahi et al., 2017). In addition, the porous structure of biochar increases soil porosity, improves soil permeability, promotes crop root development (Sadaf et al., 2017), and enhances the stability of soil aggregates (Lu et al., 2014). Meanwhile, the porous structure and surface polar functional groups of biochar enhance the adsorption of soil nutrients, thereby significantly reducing the leaching of soil nutrients (Gul and Whalen, 2016; Oliveira et al., 2017). Soil microbial biomass (MB), activity, and diversity have also been improved to different degrees after biochar application (Farrell et al., 2013; Zhang et al., 2018).

Numerous studies on the application of biochar and nitrogen to soil have been reported up to date (Oram et al., 2014; Sadaf et al., 2017; Wahi et al., 2017; Yu et al., 2018) with results showing good performance in the improvement of farmland degraded soil (Sadaf et al., 2017; Wahi et al., 2017; Li et al., 2019). However, there are few studies on using the combination of biochar and nitrogen to restore degraded grasslands, and the detailed effects need to be evaluated through empirical research. Compared with croplands, the plant diversity and the system complexity of grasslands are much higher. These differences might lead to different effects of biochar and nitrogen fertilizer on soil physicochemical properties and microorganisms of grasslands. Taking into account the ecological importance of the Qinghai-Tibet Plateau and the urgency of restoring its degradation, we took the degraded alpine grassland of the Qinghai-Tibet Plateau as the research object and used biochar and nitrogen fertilizer as additives to explore the effects of biochar and nitrogen fertilizer addition on soil physicochemical properties and microorganisms in different soil layers (0–10 and 10–20 cm) of degraded alpine grassland. We hypothesized that: (1) although grasslands were different from cropland, the addition of biochar and nitrogen fertilizer could significantly improve the soil physicochemical properties and soil microbial composition of the moderate–severe degraded grassland; (2) the improvement effect of the mixed application of biochar and nitrogen fertilizer might be better than that of biochar or nitrogen application alone.

## Materials and Methods

### Study Site

The experimental site was located in the northeast of Senduo town, Guinan county, Qinghai province (36°35′N, 101°42′E, 3220 m a.s.l.). Region climate is a typical plateau continental climate with a 2.3°C annual average temperature and 403.80 mm annual average precipitation. The annual sunshine hours are 2,738 h, and the annual evaporation is 1,378.5 mm. The main plant species in the experimental grassland include *Poa crymophila, Cleistogenes squarrosa, Carex tristachya*, *Elymus nutans. Griseb, Ligularia Cass, Stipa krylovii* Roshev., *Oxytropis kansuensis* Bunge, and *Stellera chamaejasme* Linn. The soil type is chernozem.

### Experimental Design and Soil Sampling

According to the national standard “Parameters of degradation, sandification and salification of rangelands (GB19377-2003),” alpine grasslands with 40–60% coverage and less than 10 plant species were defined as the moderate–severe degraded alpine grasslands and were selected as the research object in the middle of July 2016. The whole experimental area was fenced off from grazing disturbance in March 2017. Biochar and nitrogen fertilizer was applied in May 3, 2017. Experimental biochar was made from corn straw by pyrolysis at 550°C for 1 h. Within biochar, organic carbon was 508.9 g/kg, total nitrogen (TN) was 10.2 g/kg, total phosphorus was 80.95 g/kg, electrical conductivity was 1,595 μs/cm, and pH was 8.96. The nitrogen fertilizer used for the application was urea.

Four treatments were established, including control (without any addition, CK), biochar addition alone (2%, 4.00 kg/m^2^, C; the initial soil bulk density in the experimental plots was 1 g/cm^3^, and the percentage was the ratio of biochar weight to the dry weight of soil in the 20 cm soil surface layer), nitrogen addition alone (22 g m^–2^, urea, N), and the mixed addition of biochar and nitrogen (4.00 kg biochar/m^2^ and 22 g urea/m^2^, C + N). The amount of biochar and nitrogen application was the regular amount in agricultural practices. The experimental design was a complete random block design with three blocks. Each experimental plot was 2 m length × 2 m width. A buffer zone of 1.5 m width was set between each plot, and a 3 m width buffer strip was set between each block. The buffer zones were without any processing.

In the process of biochar and nitrogen addition, the combined method of hole application and surface application was adopted to reduce the damage to grassland and accelerate the transport of biochar particles and nitrogen in the soil. Sixteen holes (20 cm depth) were drilled in each plot with a diameter of 3.5 cm soil auger, and there was a spacing of 50 cm among holes. According to the treatment design, the mixture of biochar and nitrogen or biochar was first filled into the holes, and the remaining was evenly sprayed on the surface of the plot through the surface application. For the N treatment plot, half of the nitrogen was added to the hole and the other half was evenly sprayed on the surface through the surface application. The appropriate amount of original soil was used to cover the surface application thinly and then gently tramped to prevent the surface application from being blown away by the wind. The addition of biochar and nitrogen was conducted only in 2017.

Soil samples were collected from each plot during the peak growing period of plants (late July) in 2017, 2018, and 2019. In each plot, three sampling points were randomly selected. At each sampling point, a soil auger with a diameter of 3.5 cm was used to take soil samples from 0 to 10 cm and 10 to 20 cm depth, respectively. If we met the borehole of fertilization addition when we took soil samples, a new sample site was picked. Three soil samples from the same layer within the same sampling plot were combined into one composite sample and then separated into three parts. One part was placed into an aluminum box for soil water content (SWC) determination, one part was put in a kraft paper bag and dried at 65°C for soil physicochemical analysis, and the third part was put in a ziplock bag stored in the 0°C cooler and quickly sent back to the laboratory to store at −20°C refrigerator for soil microorganism analysis.

### Soil Physicochemical Properties Analysis

Soil physicochemical analysis included the determination of SWC, pH, electrical conductivity (EC), TOC, TN, nitrate–nitrogen (NO_3_^–^-N), ammonium nitrogen (NH_4_^+^-N), and available phosphorus (AP). SWC was calculated as follows: SWC = (W2 − W3) × 100 / (W3 − W1), where W1 was the weight of the aluminum box, W2 was the weight of the initial soil sample with the aluminum box, and W3 was the weight of the dried soil sample with the aluminum box. Soil pH and EC values were determined by the acidity meter and conductivity meter (METTLER TOLEDO, Switzerland), respectively. TOC and TN were measured by Fisher 2000 elemental analyzer (Thermo Fisher Scientific, Italy). NH_4_^+^-N and NO_3_^–^-N were determined by a flow autoanalyzer (FIA Compact, Germany). AP was measured by the sodium bicarbonate extraction molybdenum antimony anti-colorimetric method (Jinghua, China).

### Soil Microorganism Analysis

Phospholipid fatty acid (PLFA) analysis was used to determine the main microorganisms of the soil. The method described by Bligh and Dyer (1959) and modified by White et al. (1979) was used to extract lipids from 8 g of freeze-dried soil using a single-phase mixture of chloroform, methanol, and phosphate buffer (1:2:0.8). Silicic acid column chromatography removed neutral lipids and other impurities from crude extracts. Then, the purified products were saponified to get fatty acids. A moderate methyl ester method was used to methylate fatty acids to obtain phospholipid fatty acid methyl esters (FAMEs). The FAMEs were analyzed *via* gas chromatography (Agilent 6850, United States) with Sherlock MIDI software (Newark, DE, United States).

The total PLFA concentration was used to determine the total MB for both the identified (phospholipid fatty acid composition, biomass, and activity). The bacterial PLFA markers include i14:0, 14:0, a15:0, i15:0, 15:0, i16:0, 16:0, 16:1w7, a17:0, i17:0, iso17:1, 17:0, 18:1 w5, 18:1 w7, 18:0, cy17:0, and cy19:0. The abundance of the PLFAs 18:2ω6c and 18:1ω9c indicates the presence of fungal biomass. Actinomycetes are predicted by the PLFAs 10Me17:0, 10Me17:0, and 10Me18:0. The PLFAs 16:1ω7, cy17:0, and cy19:0 are considered to be markers of Gram-negative bacteria (Gn). The PLFAs i14:0, a15:0, i15:0, i16:0, i17:0, and a17:0 are considered to be markers of Gram-positive bacteria (Gp) (Frostegard and Baath, 1996; Evgrafova et al., 2008; Djukic et al., 2010). Individual PLFA markers are as follows: 15:0iso is a marker of Gram-positive bacteria, 16:1w7c is a marker of Gram-negative bacteria, 16:10 methyl is a marker of anaerobic bacteria (Ab), 16:1w5c is a marker of arbuscular mycorrhizal fungi (Am), 18:1w7c is a marker of methanotrophic bacteria (Mb), 18:1w9c is a marker of ectomycorrhizal fungi (Ef), 18:2w6,9c is a marker of saprotrophic fungi (Sf), and 19:0 cyclo is a marker of anaerobic bacteria (An) (Sundh et al., 2000; Smith et al., 2015; Li et al., 2020).

### Statistical Analysis

One-way ANOVA and Tukey’s *post hoc* test (with a confidence of 95%) were used to analyze the differences between treatments in different years. Soil property and PLFA measurements were indicated by the mean of three replicates with the standard error (SE) of the mean. Principal component analysis (PCA) was used to compare and analyze the structure of the soil microbe community, which was indicated by the composition of PLFAs. The relationships between soil physicochemical properties and special microorganisms were analyzed using redundant analysis (RDA). The correlation of soil physicochemical was analyzed using Pearson’s correlation, and the Mantel test was used to explore the relationship between different microbial communities and environmental factors in different treatments. Procrustes analysis of the correlation between soil physicochemical properties and microbial community was based on the PCA (Bray–Curtis) results of different treatments. All analyses were performed in R3.3.3. Effects were considered to be significant if *P* < 0.05.

## Results

### Changes of Soil Physicochemical Properties in Moderate–Severe Degraded Alpine Grassland

In the 0–10 cm soil layer, pH had no significant change among treatments in 2017, but pH was significantly higher in the treatments of biochar application than that in CK in 2018 and 2019. In the 10–20 cm soil layer, pH was significantly higher in treatments with additives than that in CK (Table 1). In the first experimental year (2017), EC in the 0–20 cm soil layer was much higher in the treatments of N addition, biochar addition, and the mixed addition of nitrogen and biochar than that in CK. In the third year (2019), EC within the treatments of biochar addition dropped to a similar value to that in CK, but EC in the treatment with N addition dropped to a value smaller than that in CK (Table 1). SWC in the 0–20 cm soil layer did not change significantly among different treatments in 2017, but was significantly higher in the treatments with additives than that in CK in 2018 and 2019 (Table 1). SWC in the 10–20 cm soil layer showed a similar trend in the surface layer.

**TABLE 1:** Variation of soil pH, electrical conductivity (EC), and soil water content (SWC) in the 0–20 cm soil layers under different treatments in 2017, 2018, and 2019.

Parameter	Year	Layer (cm)	CK	N	C	C + N
pH	2017	0–10	7.12 ± 0.06 aB	7.31 ± 0.09 aA	7.36 ± 0.15 aB	7.25 ± 0.05 aC
	2018		7.55 ± 0.04 bA	8.00 ± 0.6 abA	8.22 ± 0.26 aA	8.43 ± 0.24 aA
	2019		7.28 ± 0.27 bAB	7.66 ± 0.08 abA	7.75 ± 0.06 aA	7.78 ± 0.21 aB
	2017	10–20	7.24 ± 0.13 aA	7.35 ± 0.21 aB	7.31 ± 0.10 aC	7.38 ± 0.09 aB
	2018		7.48 ± 0.11 bA	8.26 ± 0.28 aA	8.44 ± 0.10 aA	8.42 ± 0.25 aA
	2019		7.49 ± 0.06 bA	8.25 ± 0.28 aA	7.81 ± 0.14 abB	7.99 ± 0.23 abA
EC (us/cm)	2017	0–10	362.76 ± 56.72 bB	430 ± 22.52 abA	502 ± 25.53 aA	483.67 ± 46.19 aA
	2018		574.09 ± 34.95 aA	129.79 ± 50.7 cB	302.58 ± 38.66 bB	297.92 ± 18.98 bB
	2019		209.80 ± 25.97 aC	109.79 ± 50.7 bB	220.73 ± 33.03 aB	222.93 ± 33.39 aB
	2017	10–20	324.91 ± 13.73 bB	423 ± 26.89 aA	419 ± 19.67 aA	411.33 ± 45.8 aA
	2018		605.27 ± 121.87 aA	107.65 ± 9.52 cB	282.62 ± 19 bB	342.53 ± 79.77 abA
	2019		197.53 ± 17.65 aB	97.65 ± 9.52 bB	198.33 ± 28.29 aB	195.2 ± 7.7 aB
SWC (%)	2017	0–10	24.52 ± 1.13 aA	24.85 ± 1.12 aB	24.35 ± 1.01 aB	25.76 ± 1.59 aAB
	2018		18.82 ± 0.47 bB	30.87 ± 3.26 aA	30.69 ± 2.22 aA	30.84 ± 3.06 aA
	2019		16.46 ± 1.24 bB	23.54 ± 0.63 aB	27.01 ± 3.26 aB	25.43 ± 0.75 aB
	2017	10–20	21.14 ± 0.87 aA	22.68 ± 2.34 aAB	21.89 ± 1.02 aB	21.25 ± 0.48 aB
	2018		19.85 ± 0.62 bA	25.85 ± 1.49 aA	27.31 ± 0.92 aA	28.61 ± 1.90 aA
	2019		16.08 ± 1.00 cB	19.01 ± 2.13 bB	20.55 ± 1.21 abB	25.85 ± 1.49 aA

*Values are means ± SE, lowercase letters indicate differences between different treatments in the same year (P < 0.05), and uppercase letters indicate differences between different years in the same treatment (P < 0.05).*

**FIGURE 1:**
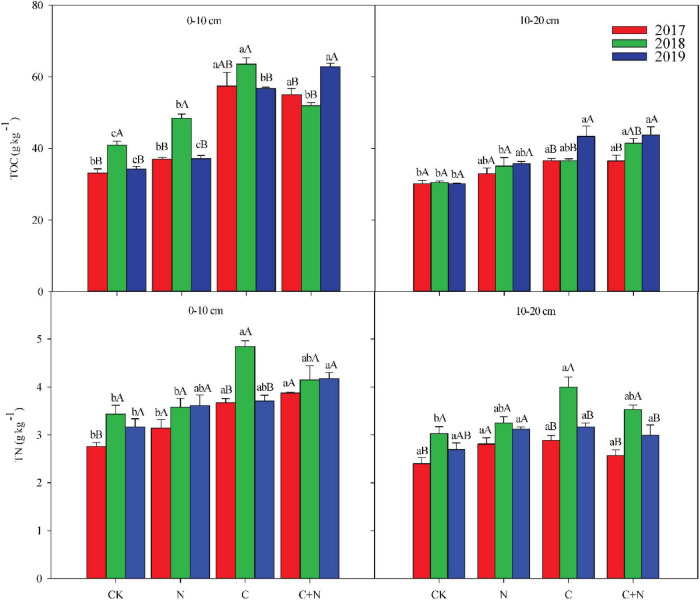
Effects of different treatments on soil total organic carbon (TOC) and soil total nitrogen (TN) in 0–20 cm soil layers in 2017, 2018, and 2019. Error bars represent standard errors of the mean values, and different lowercase letters over the bars indicate differences among different treatments in the same year at *P* < 0.05. Different uppercase letters over the bars indicate differences among different years in the same treatment at *P* < 0.05.

The addition of biochar and nitrogen significantly changed TOC and TN in the 0–20 cm soil layer (Figure 1). Compared with CK, the addition of nitrogen alone had no significant effect on the TOC in the 0–10 cm soil layer, but the addition of biochar and the mixed addition of biochar and nitrogen significantly increased the TOC in the topsoil (0–10 cm). In the third experimental year (2019), TOC in the 0–10 cm soil layer reached 62.79 g/kg in the treatment of the mixed addition of biochar and nitrogen. In the 10–20 cm soil layer, the addition of N alone did not increase the TOC compared with CK, but the addition of biochar significantly increased the TOC and showed an upward trend year by year (Figure 1). In the third experimental year (2019), the TOC increased by 43.58 and 45.07% in the treatments of biochar addition and the mixed addition of nitrogen and biochar, respectively, compared with CK (Figure 1). TN in the topsoil (0–10 cm) increased significantly under the treatments of biochar addition and the mixed addition of nitrogen and biochar. Compared with nitrogen addition alone, the addition of biochar and the mixed addition of nitrogen and biochar resulted in higher TN accumulation in the topsoil (Figure 1). Ratio of carbon to nitrogen (C/N) in the 0–20 cm soil layer was higher in the treatments with biochar addition than that in CK, but C/N in the treatment with nitrogen addition alone showed no significant difference with CK (Table 2).

**TABLE 2:** Variation of soil ammonia-nitrogen (NH_4_^+^-N), nitrate-nitrogen (NO_3_^–^-N), available phosphorus (AP), and the ratio of carbon to nitrogen (C/N) in the 0–20 cm soil layers under different treatments in 2017, 2018, and 2019.

Parameter	Year	Layer (cm)	CK	N	C	C + N
NH_4_^+^-N (mg/kg)	2017	0–10	17.5 ± 0.25 aC	18.12 ± 1.81 aB	11.74 ± 0.11 bC	16.8 ± 1.13 aC
	2018		35.05 ± 4.49 bA	78.83 ± 9.53 aA	70.37 ± 8.26 aA	66.64 ± 3.67 aA
	2019		26.13 ± 1.47 cB	48.83 ± 9.53 aB	48.35 ± 2.90 bB	45.38 ± 2.07 cB
	2017	10–20	16.71 ± 1.84 aB	11.38 ± 1.78 bB	9.65 ± 0.53 bB	9.39 ± 0.66 bB
	2018		22.17 ± 1.61 bA	36.52 ± 5.21 aA	34.56 ± 3.3 aA	41.96 ± 6.94 aA
	2019		14.64 ± 2.59 bB	20.53 ± 5.21 aB	36.06 ± 3.11 aA	36.50 ± 5.52 bB
NO_3_^–^-N (mg/kg)	2017	0–10	7.57 ± 1.91 bA	18.5 ± 1.95 aB	21.62 ± 1.54 aB	21.76 ± 1.89 aB
	2018		4.97 ± 0.85 bA	9.57 ± 1.78 aC	5.77 ± 1.33 bC	9.09 ± 0.18 aC
	2019		8.96 ± 2.11 bA	26.57 ± 1.78 bA	31.7 ± 6.39 aA	36.73 ± 2.84 aA
	2017	10–20	7.18 ± 1.33 bAB	8.72 ± 0.56 bB	13.29 ± 1.36 aA	12.89 ± 2.44 aA
	2018		4.94 ± 0.97 cB	11.16 ± 0.7 aA	8.01 ± 1.61 bB	8.87 ± 0.98 bB
	2019		11.09 ± 3.66 aA	12.66 ± 0.7 aA	13.79 ± 1.36 aA	13.22 ± 1.67 aA
AP (mg/kg)	2017	0–10	2.58 ± 0.29 bA	2.62 ± 0.5 bA	4.19 ± 0.52 aA	4.63 ± 0.12 aA
	2018		3.02 ± 0.29 bA	3.10 ± 0.26 bA	4.37 ± 0.09 aA	4.42 ± 0.37 aA
	2019		1.57 ± 0.21 bB	3.10 ± 0.26 aA	3.31 ± 0.12 aA	3.49 ± 0.49 aA
	2017	10–20	1.79 ± 0.16 cA	2.24 ± 0.23 bcA	3.12 ± 0.3 aA	2.64 ± 0.2 abAB
	2018		1.89 ± 0.44 cA	2.24 ± 0.05 bcA	2.72 ± 0.15 abAB	3.16 ± 0.35 aA
	2019		1.49 ± 0.03 bA	2.14 ± 0.07 aA	2.37 ± 0.29 aB	2.20 ± 0.12 aB
C/N	2017	0–10	12.02 ± 0.6 bA	12.09 ± 1.16 bA	15.63 ± 1.66 aA	14.21 ± 0.7 abA
	2018		12.02 ± 1.69 abA	10.36 ± 1.35 bA	13.13 ± 0.72 aA	13.67 ± 1.16 aA
	2019		10.85 ± 0.7 bA	9.29 ± 1.24 bA	15.34 ± 0.71 aA	15.22 ± 1.19 aA
	2017	10–20	12.68 ± 1.69 bA	11.80 ± 1.42 bA	12.73 ± 1.09 bA	14.27 ± 1.74 aA
	2018		10.08 ± 0.58 abA	9.81 ± 0.99 bA	9.22 ± 1.00 bB	11.76 ± 0.58 aB
	2019		10.03 ± 0.97 abA	10.33 ± 0.84 bA	13.67 ± 1.22 aA	11.78 ± 1.86 bB

*Values are means ± SE, lowercase letters indicate differences between different treatments in the same year (P < 0.05), and uppercase letters indicate differences between different years in the same treatment (P < 0.05).*

NH_4_^+^-N in the 0–10 cm soil layer had no significant difference among treatments in the first year (2017), but was significantly greater in the treatments with additives in the second and third years (2018 and 2019, respectively) than that in CK (Table 2). Within the same treatment, NH_4_^+^-N increased first and then decreased with the increase of experimental years (Table 2). In the 10–20 cm soil layer, the changes of NH_4_^+^-N in each treatment or between treatments were similar to that in the topsoil. Different from the changing trend of NH_4_^+^-N, NO_3_^–^-N in the 0–10 cm soil layer was significantly greater in the treatments with additives than that in CK. Within the same treatment, NO_3_^–^-N first decreased and then increased with the increase of the experimental year. The maximum value appeared in the third year (2019). In the 10–20 cm soil layer, NO_3_^–^-N was greater in the treatments with additives than that in CK in the first 2 years (2017 and 2018), but in the third experimental year (2019), NO_3_^–^-N had no significant difference between CK and the treatments with additives (Table 2). Compared with CK, AP in the 0–20 cm soil layer increased in the treatments with additives. Among these treatments, the addition of biochar and the mixed addition of biochar and nitrogen had a stronger effect on AP increase. AP in the third experimental year (2019) was relatively lower than that in 2017 and 2018 (Table 2).

### Changes of Soil Microorganisms in the Moderate–Severe Degraded Alpine Grassland

**FIGURE 2:**
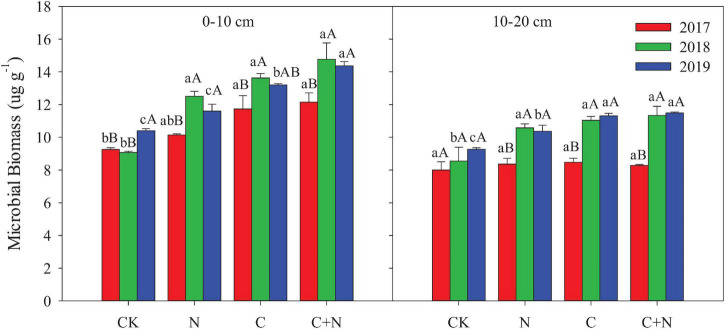
Effects of different treatments on bacteria and fungi in 0–20 cm soil layers in 2017, 2018, and 2019. Error bars represent standard errors of the mean values, and different lowercase letters over the bars indicate differences among different treatments in the same year at *P* < 0.05. Different uppercase letters over the bars indicate differences among different years in the same treatment at *P* < 0.05.

Compared with CK, the N addition alone had no significant effect on the MB in the 0–10 cm soil layer, but the addition of biochar and the mixed addition of biochar and nitrogen increased the MB (Figure 2). With the increase of experimental year, the MB showed a certain upward trend in the treatments with additives, especially in the treatment of the mixed addition of biochar and nitrogen. The MB increased from 12.15 μg/g in 2017 to 14.37 μg/g in 2019 in the treatment of the mixed addition of biochar and nitrogen. In the 10–20 cm soil layer, the MB had no significant difference among treatments in the first experimental year (2017), but the treatments with additives had higher MB in the second and third years (2018 and 2019, respectively) than CK (Figure 2). For the same treatment, MB increased with the increase of experimental year.

**FIGURE 3:**
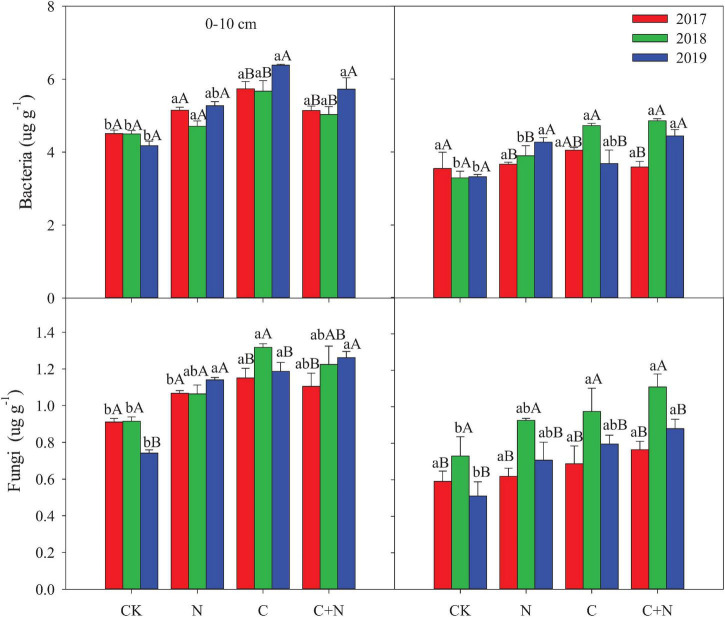
Effects of different treatments on microbial biomass in 0–20 cm soil layers in 2017, 2018, and 2019. Error bars represent standard errors of the mean values, and different lowercase letters over the bars indicate differences among different treatments in the same year at *P* < 0.05. Different uppercase letters over the bars indicate differences among different years in the same treatment at *P* < 0.05.

Bacteria biomass in the topsoil (0–10 cm) increased in the treatments with additives compared with CK, and the increased intensity was greater in the addition of biochar and the mixed addition of biochar and nitrogen than that in the nitrogen addition alone. In the 10–20 cm soil layer, treatments with additives had a higher bacterial biomass than CK in the second and third experimental years (2018 and 2019, respectively), and the mixed addition of biochar and nitrogen reached the highest value (Figure 3). Similar to the changes of bacteria, fungi in the 0–10 cm soil layer was significantly greater in the treatments with additives than that in CK, and the increased intensity was greater in the addition of biochar and the mixed addition of biochar and nitrogen than that in the nitrogen addition. In the 10–20 cm soil layer, fungi had no significant difference among treatments in the first experimental year (2017), but the treatments with additives increased the relative content of fungi of this layer compared with CK in the second and third experimental years (Figure 3). The fungi:bacteria (F/B) in the 0–10 cm soil layer was greater in the treatments with additives than that in CK. In the 10–20 cm soil layer, the N addition alone did not have a significant effect on the F/B, but the F/B increased in the treatment of biochar addition alone and the mixed addition of biochar and nitrogen (Table 3). Compared with CK, MB:TOC in the 0–10 cm soil layer decreased in the treatments of biochar addition alone and the mixed addition of biochar and nitrogen, but had no change in the treatment of N addition alone.

**TABLE 3:** Fungi:bacteria (F/B), Gram-positive:Gram-negative (GP/GN), microbial biomass:total organic carbon (MB:TOC), and microbial biomass:total nitrogen (MB:TN) in 0–20 cm soil layers under different treatments in 2017, 2018, and 2019.

Parameter	Year	Layer (cm)	CK	N	C	C + N
F/B	2017	0–10	0.18 ± 0.01 bA	0.21 ± 0 aA	0.22 ± 0.02 aB	0.22 ± 0.04 aA
	2018		0.20 ± 0.01 aA	0.20 ± 0.01 aA	0.25 ± 0.01 aA	0.22 ± 0.01 aA
	2019		0.17 ± 0.01 aA	0.19 ± 0.02 aA	0.21 ± 0.01 aB	0.20 ± 0.01 aA
	2017	10–20	0.17 ± 0.01 bB	0.17 ± 0.01 bA	0.21 ± 0.07 aB	0.18 ± 0.04 bB
	2018		0.22 ± 0.03 bA	0.19 ± 0.01 bA	0.28 ± 0.05 bA	0.31 ± 0.15 aA
	2019		0.18 ± 0.02 aB	0.16 ± 0.03 abA	0.26 ± 0.01 abA	0.25 ± 0.04 bA
GP/GN	2017	0–10	1.44 ± 0.14 aAB	1.36 ± 0.11 aA	1.37 ± 0.04 aA	1.46 ± 0.05 aA
	2018		1.55 ± 0.06 aA	1.40 ± 0.24 abA	1.41 ± 0.2 abA	1.33 ± 0.01 bB
	2019		1.29 ± 0.06 aB	1.28 ± 0.04 aB	1.13 ± 0.01 bB	1.27 ± 0.02 aB
	2017	10–20	1.12 ± 0.23 aA	1.17 ± 0.02 aA	1.24 ± 0.10 aA	1.23 ± 0.15 aA
	2018		1.19 ± 0.04 bA	1.20 ± 0.13 bA	1.23 ± 0.20 bA	1.56 ± 0.13 aA
	2019		1.11 ± 0.10 aA	1.15 ± 0.05 aA	1.12 ± 0.06 aA	1.07 ± 0.01 aB
MB:TOC	2017	0–10	0.28 ± 0.01 aA	0.27 ± 0.00 aA	0.21 ± 0.02 bA	0.22 ± 0.02 bA
	2018		0.22 ± 0.01 bB	0.26 ± 0.01 abA	0.21 ± 0.01 bA	0.23 ± 0.03 bA
	2019		0.31 ± 0.01 aA	0.31 ± 0.03 aA	0.22 ± 0.00 bA	0.23 ± 0.01 bA
	2017	10–20	0.27 ± 0.04 aA	0.25 ± 0.01 aA	0.23 ± 0.01 aB	0.23 ± 0.01 aA
	2018		0.28 ± 0.04 aA	0.31 ± 0.04 aA	0.30 ± 0.01 aA	0.27 ± 0.01 aA
	2019		0.31 ± 0.00 aA	0.29 ± 0.01 aA	0.26 ± 0.03 aAB	0.26 ± 0.02 aA
MB:TN	2017	0–10	3.36 ± 0.11 aA	3.25 ± 0.22 aA	3.20 ± 0.39 aA	3.14 ± 0.22 aB
	2018		2.65 ± 0.19 bB	3.14 ± 0.4 abA	2.82 ± 0.05 bB	3.89 ± 0.46 aA
	2019		3.35 ± 0.25 aA	2.91 ± 0.38 bA	3.57 ± 0.17 aA	3.48 ± 0.11 abA
	2017	10–20	3.34 ± 0.16 aA	2.98 ± 0.14 aA	2.94 ± 0.20 aAB	3.24 ± 0.23 aA
	2018		2.84 ± 0.45 aA	2.98 ± 0.32 aA	2.78 ± 0.24 aB	3.22 ± 0.23 aA
	2019		3.50 ± 0.27 abA	2.91 ± 0.19 bA	3.58 ± 0.2 abA	3.89 ± 0.42 aA

*Values are means ± SE, lowercase letters indicate differences between different treatments in the same year (P < 0.05), and uppercase letters indicate differences between different years in the same treatment (P < 0.05).*

**FIGURE 4:**
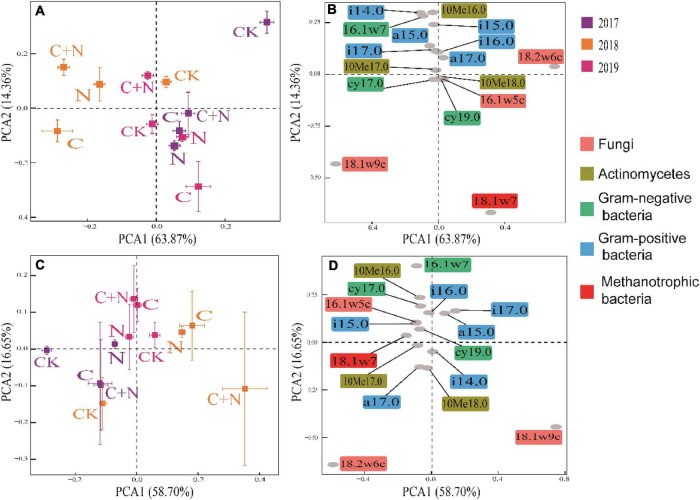
Principal component analysis (PCA) of soil microbial community within different soil layers under different treatments in 2017, 2018, and 2019. **(A)** Treatment coordinates of 0–10 cm soil layer; **(B)** soil microorganism coordinates of 0–10 cm soil layer; **(C)** treatment coordinates of 10–20 cm soil layer; and **(D)** soil microorganism coordinates of 10–20 cm soil layer.

In PCA, the first two axes explained the variation of 78.23% in the 0–10 cm soil layer (Figure 4A) and 75.35% in the 10–20 cm soil layer (Figure 4C), respectively. The dispersion degree of the treatments among different years in the coordinate axis was greater in the 0–10 cm soil layer than that in the 10–20 cm soil layer, and the dispersion degree of different treatments increased with the increase of experimental year. It indicated that the addition of biochar and nitrogen had a significant impact on the microbial community structure of the topsoil, and the degree of influence became greater with the increase of residence time in soil, especially for the treatment of biochar addition alone and the mixed addition of biochar and nitrogen (Figures 4A,C). Compared with CK, biochar addition alone significantly increased the relative content of 16.1w5c, cy17.0, cy19.0, 10Me18.0, 18.1w7, and 18.1w9c, the mixed addition of biochar and nitrogen significantly increased the relative content of i14.0, i15.0, a15.0, 10Me16.0, 16.1w7, i16.0, i17.0, 10Me17.0, cy17.0, and 18.1w9c, but the nitrogen addition alone did not cause the significant change of soil microbial community in the topsoil (0–10 cm) (Figures 4A,B). In the deep soil (10–20 cm), the differences of microbial community structure among treatments were mainly reflected in experimental years, and the treatments of biochar addition alone and the mixed addition had figured prominently. Compared with CK, the relative contents of i14.0, a17.0, 10Me18.0, and 18.1w9c in 2017 were greater in the treatments of biochar addition alone and the mixed addition; the relative contents of a15.0, i17.0, and 18.1w9c were greater in 2018; and the relative contents of other microorganisms, except for i14.0, a17.0, 10Me18.0, and 18.1w9c, were greater in 2019 (Figures 4C,D). Among these microorganisms, 18.1w9c, 18.2w6c, and 18.1w7 had greater contributions (Figure 4).

### The Interaction Between Microorganisms and Soil Properties

**FIGURE 5:**
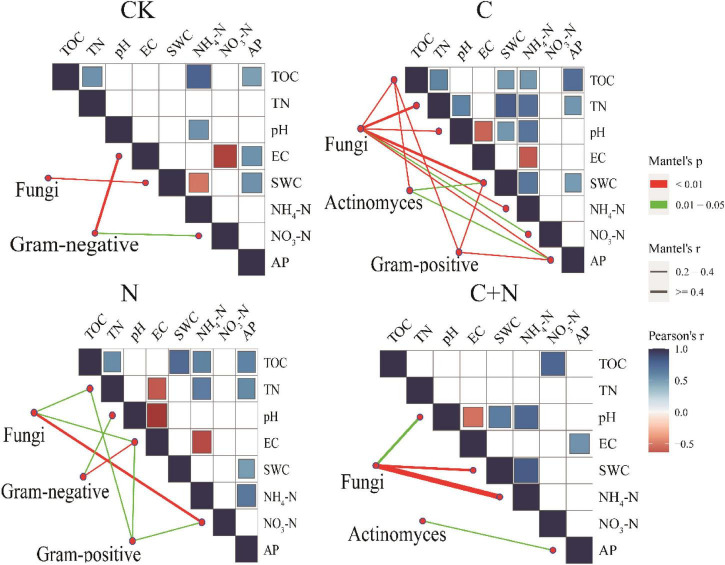
Pairwise comparisons between environmental factors and microbial communities in the 0–10 cm and 10–20 cm soil layers by partial Mantel tests for different treatments. The color gradients denote Spearman’s correlation coefficients; line edge width corresponds to the Mantel’s *r* statistic for the corresponding distance correlations, and edge color denotes the statistical significance based on 9,999 permutations.

**FIGURE 6:**
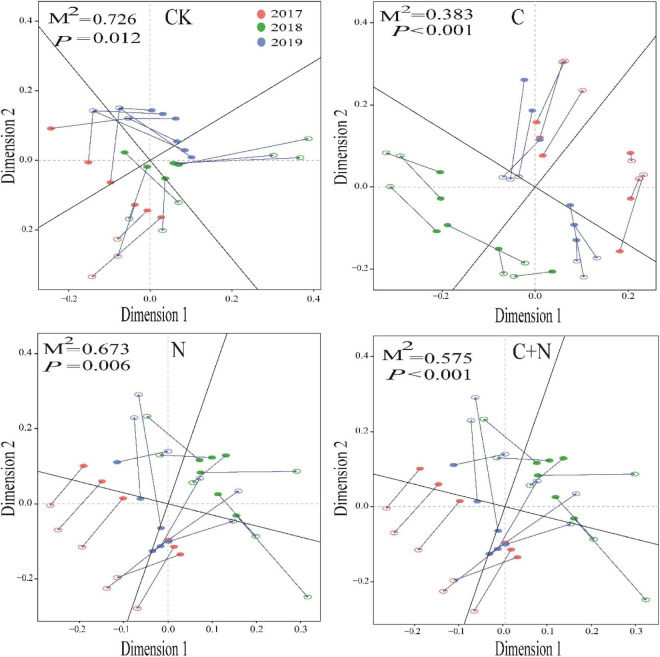
Procrustes analysis of the correlation between soil physicochemical properties and microbial community based on the PCA (Bray–Curtis) results of different treatments.

Different treatments showed various interactions between microorganisms and soil physicochemical properties. In CK, the interactions between microorganisms and soil physicochemical properties were weak, and only fungi and SWC, Gram-negative, EC, and NO_3_^–^-N had a strong interaction (Figure 5). In the treatment of nitrogen addition alone, fungi had significant interactions with TN, EC, and NO_3_^–^-N (Figure 5). In the treatment of biochar addition alone, most soil physicochemical properties showed significant positive correlations, and interactions between soil factors and fungi, actinomyces, and Gram-positive had improved. Especially, fungi had a significant interaction with most of the soil factors (Figure 5). In the treatment of the mixed addition, fungi showed significant correlations with pH, SWC, and NH_4_^+^-N (Figure 5). The Procrustes analysis further verified the potential relationship between soil factors and microbial community under different treatments (Figure 6). It showed that the *P*-values of different treatments in the Procrustes analysis were all less than 0.05, and the performance of *M*^2^ was C (*M*^2^ = 0.383) < C+N (*M*^2^ = 0.575) < N (*M*^2^ = 0.673) < CK (*M*^2^ = 0.726) (Figure 6). It indicated that the consistency of the relationship between soil factors and microbial community was greater in the treatment of biochar addition alone and the mixed addition than that in the treatment of nitrogen addition alone and CK, and the biochar addition alone and the mixed addition had greater improving effect on the interaction between microorganisms and main soil physicochemical properties.

**FIGURE 7:**
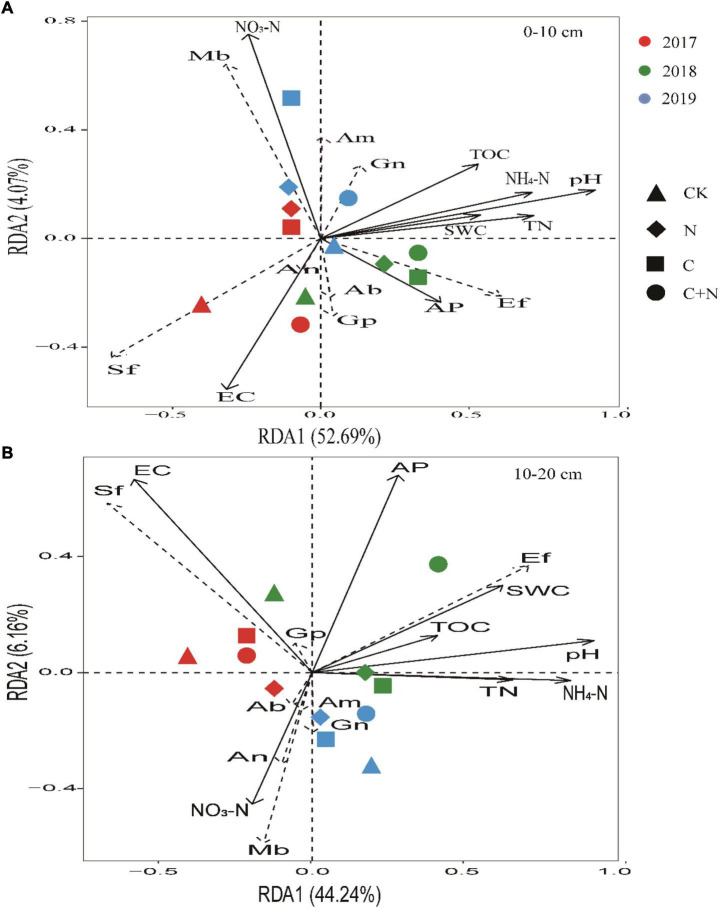
Redundancy analysis (RDA) ordination biplot of soil physicochemical properties and microbes in 0–10 cm soil layer **(A)** and 10–20 cm soil layer **(B)**. Soil physicochemical properties were represented by black lines, and microorganism indicators were represented by dashed lines. TOC, total organic carbon; TN, total nitrogen; AP, available phosphorus; NO_3_^–^-N, soil nitrate–nitrogen; NH_4_^+^-N, soil ammonia–nitrogen; EC, electrical conductivity; SWC, soil water content. Gn, Gram-negative bacteria; Ab, Actinobacteria; Gp, Gram-positive bacteria; Sf, saprotrophic fungi; Ef, ectomycorrhizal fungi; Mb, methanotrophic bacteria; An, anaerobic bacteria; Am, arbuscular mycorrhizal fungi.

Focused on the relationship between soil physicochemical properties and specific microbes, RDA found that NO_3_^–^-N, EC, and pH in the topsoil (0–10 cm) had a greater effect on specific microbial community structures. Ef and Gn had a positive correlation with most of the soil properties. Sf had a positive correlation with EC, and Mb had a positive correlation with NO_3_^–^-N. With the increase of experimental year, the sensitivity of microorganisms to soil properties significantly increased in the treatments of biochar addition alone and the mixed addition (Figure 7A). In the 10–20 cm soil layer, EC, AP, and pH had a greater effect on specific microbial community structures (Figure 7B). Ef had a positive correlation with most of the soil properties. Sf had a positive correlation with EC, and Mb had a positive correlation with NO_3_^–^-N.

## Discussion

### Responses of Soil Physicochemical Properties to the Addition of Biochar or Nitrogen

When additives were added to the soil, it generally changed the physicochemical properties of the soil to some extent. In this study, soil TOC and TN significantly increased after the addition of biochar and the mixed addition of biochar and nitrogen, and the amplitude of increase was greater in the 0–10 cm soil layer than that in the 10–20 cm soil layer. Moreover, TOC and TN continuously increased with the increase of residence time of additives in soil (Figure 1). The accumulation of TOC in soil was probably associated with the unique properties of biochar. On the one hand, biochar has the capacity of strong adsorption to adsorb small organic molecules in the soil. These small organic molecules can polymerize to form organic matter under the catalysis, and then the soil organic carbon increases (Muhammad et al., 2018). On the other hand, biochar contains a large amount of organic carbon with an aromatic structure, which is considered as the recalcitrant carbon pool. Therefore, biochar addition increases the accumulation of soil organic carbon (Chen et al., 2018). In addition, MB also increased significantly after biochar addition (Figure 2). The increase of microorganisms can accelerate the decomposition of organic matter and lead to the increase of organic carbon (Pietikainen et al., 2000). The unique properties of biochar also promoted the increase of TN in soil. A study found that the cation exchange capacity of biochar increased as the residence time of biochar in the soil increased, then the holding capacity of nutrients was strengthened, and the leaching of soil nutrients decreased (Jiang et al., 2019), and therefore, the accumulation of nitrogen in the 0–20 cm soil layer increased. Biochar addition increased the contents of trace elements, boron and manganese, related to nitrogenase activity in the soil and then improved the efficiency of nitrogen fixation (Quilliam et al., 2012). However, TOC and TN had no significant change under the treatment of nitrogen addition alone in this study. It indicated that this application method and the amount of nitrogen fertilizer did not cause an impact on soil TOC and TN of the moderate–severe degraded alpine grassland in this short-term.

The addition of biochar and nitrogen fertilizer significantly changed the content of available nitrogen NH_4_^+^-N and NO_3_^–^-N in the soil, and the effect increased with the increase of application time (Table 1). As seen from the research results, NO_3_^–^-N in soil was lower in 2018 than that in 2017 and 2019. This might be related to the higher soil pH and SWC in 2018 (Table 1). High water content causes soil hypoxia (Chen et al., 1997). Together with high pH, it strengthened the denitrification in the soil, and thereby accelerated the consumption of NO_3_^–^-N and decreased the content of NO_3_^–^-N. In addition, biochar has relatively high CEC, which can not only increase the adsorption of NH_4_^+^ but also reduce the conversion to NO_3_^–^ (Ye et al., 2020). In general, the contents of available nitrogen NH_4_^+^-N and NO_3_^–^-N were greater in the treatments of biochar addition alone and the mixed addition. It indicated that biochar addition increased the availability of nitrogen in degraded alpine grassland. Polyporous structure and strong adsorption properties of biochar also increase the absorption of phosphorus and promote the increase of soil water-holding capacity, thus increasing AP and SWC (Han et al., 2016; Reed et al., 2017). The results of this study were consistent with this finding (Tables 1, 2).

Biochar is alkaline because it forms a certain amount of carbonate during the process of preparation (Fan et al., 2018). When biochar is applied to the soil, the soil pH generally increases. The change of pH in this study verified the trend. However, the long-term excessive application of N fertilizer will lead to a decrease in pH (Fu and Shen, 2017). In this study, nitrogen fertilizer is not used excessively, so the pH had no significant change in the treatment of nitrogen addition alone. The ratio of total soil carbon to nitrogen is an important indicator to evaluate the composition and quality of soil organic matter (Fang et al., 2019). In this study, C/N increased significantly in the treatments of biochar addition alone and the mixed addition and was higher than the global average of grassland soil (11.8) (Wang X. G. et al., 2020). It indicated that biochar addition makes soil carbon be supplemented.

### Response of Soil Microorganisms to the Addition of Biochar or Nitrogen

Total MB, bacteria, and fungi in the 0–20 cm soil layer significantly increased under the addition of biochar and the mixed addition of biochar and nitrogen and increased with the increase of application time (Figures 2, 3). These changes were probably attributed to the improvement of soil properties (i.e., soil bulk density, water content, and soil nutrients) after biochar input. This improvement can provide suitable habitat for the growth and reproduction of microorganisms (Zhu et al., 2017). Furthermore, the porosity structure of biochar can absorb nutrient substrates for the growth and metabolism of microorganisms (Pietikainen et al., 2000). Nitrogen addition directly provides nutrients for the growth and reproduction of soil microorganisms (Ma et al., 2020).

Fungi:bacteria had significant differences among treatments (Table 3), but Gram-positive:Gram-negative (GP/GN) had no change. It indicated that the addition of biochar and nitrogen caused an asynchronous increase of soil bacteria and fungi and tends to increase more fungi. This finding was consistent with the findings of Zong and Fu (2021), who found that the fungal community is sensitive to soil degradation and varied in different degraded alpine grasslands. MB:TOC was greater in the treatments of biochar addition alone and the mixed addition of biochar and nitrogen, but MB:TN had no significant change among treatments (Table 3). It showed that additives with biochar were more likely to cause changes in the structure of the soil microbial community, but only nitrogen addition had a relatively weak effect. In the first year of the study, the community structure of soil microorganisms changed slightly, and the amplitude of change was greater in the surface soil (0–10 cm) than that in the deep soil (10–20 cm). In the second and third experimental years, different additive treatments caused different changes in the microbial communities. This response was probably associated with the way of “hole application” and “surface application.” This addition method might result in an insufficient mixture of the added materials with the soil in the first year and required some time to expand the added materials into the deep soil. Therefore, the difference among treatments mainly occurred in the second and third experimental years. Under the treatment of biochar addition alone, 18.1w9c and 18.2w6c had significant changes. Under the treatment of the mixed addition of biochar and nitrogen, bacteria, 16.1w5c, 18.1w9c, and 18.2w6c had significant changes. Biochar input increased the accumulation of nutrients (Figure 1 and Table 1), but biochar is rich in organic carbon and lacks other nutrients, and its input may cause the imbalance of nutrients for microbial growth over a period of time (Jenkins et al., 2017). Fungi, the main decomposer of the soil, can release extracellular enzymes to effectively degrade the stubborn organic matter components (DeForest and Snell, 2020). The input of biochar with porosity structure provides a good habitat for fungi (Zhu et al., 2017) and promotes the reproduction of fungi to ensure the accumulation of nutrients required for vegetation and microbial growth (Zhang et al., 2018). Nitrogen fertilizer, as the most direct nutrient substance, can directly provide nutrients for the growth of microorganisms (Li et al., 2018) and accelerate their reproduction (Zhao Z. B. et al., 2019).

In Procrustes analysis, the smaller value of *M*^2^ means the better consistency between environmental factors and microbial community structure (Zhao R. et al., 2019). In this study, the values of *M*^2^ in the treatments of biochar addition alone and the mixed addition were relatively higher (Figure 7). This further proved that the addition of biochar and the mixed addition improved soil microbial community and physicochemical properties at the same time. As for the addition of nitrogen alone, it produced similar results to CK. It was probably due to the volatilization or leaching of nitrogen as nitrogen was added alone (Zhu et al., 2018). This difference indirectly proved that the mixed addition of nitrogen and biochar could enhance the utilization efficiency of nitrogen fertilizer. In a degraded grassland, nitrogen is an important limiting factor for the development of soil microorganisms (Liu et al., 2020). Nitrogen input could decrease the high C/N and promote the activity of decomposers.

Biochar input could provide organic matter and absorb nutrients for the utilization of microorganisms. Also, the addition of nitrogen can quickly make up for the shortcomings of insufficient soil nutrients and stimulate the growth of microorganisms, and the addition of biochar can improve the utilization efficiency of nitrogen fertilizer and provide a good habitat for microorganisms. This was the important reason why the mixed addition of biochar and nitrogen caused higher improvement effects for the moderate–severe degraded alpine grassland.

## Conclusion

Compared with nitrogen addition alone, the addition of biochar alone and the mixed addition of biochar and nitrogen had a greater improvement effect on the soil physicochemical properties and microorganisms of the vegetation growth layer (0–20 cm). In the 0–20 cm soil layer, TOC, TN, NO_3_^–^-N, NH_4_^+^-N, AP, SWC, and MB increased significantly after the addition of biochar and the mixed addition of biochar and nitrogen. As for specific microbes, the relative content of 16.1w5c, 18.1w9c, 18.2w6c, and bacteria increased significantly as well. Moreover, the biochar addition alone or the mixed addition of biochar and nitrogen changed the structure of the microbial community and the relationships between microorganisms and soil physicochemical properties. The mixed addition of biochar and nitrogen produced the superposition effect and made it superior to the biochar addition alone in some aspects. These findings indicated that the application of biochar in the moderate–severe degraded alpine grassland could improve soil properties and microbial community structure, and the mixed addition of biochar and nitrogen had a stronger improvement effect on the soil system of moderate–severe degraded alpine grassland.

## Data Availability Statement

The original contributions presented in the study are included in the article/supplementary material, further inquiries can be directed to the corresponding author.

## Author Contributions

JL, YZ, and KL contributed to conception and design of the study. YZ, HL, and YH organized the database. JL performed the statistical analysis. JL and YZ wrote the first draft of the manuscript. XS, DH, JS, and KL wrote sections of the manuscript. All authors contributed to manuscript revision, read, and approved the submitted version.

## Conflict of Interest

The authors declare that the research was conducted in the absence of any commercial or financial relationships that could be construed as a potential conflict of interest.

## Publisher’s Note

All claims expressed in this article are solely those of the authors and do not necessarily represent those of their affiliated organizations, or those of the publisher, the editors and the reviewers. Any product that may be evaluated in this article, or claim that may be made by its manufacturer, is not guaranteed or endorsed by the publisher.

## Abbreviations

TOC: total organic carbon
TN: total nitrogen
SWC: soil water content
EC: electrical conductivity
AP: available phosphorus
C/N: ratio of carbon to nitrogen
F/B: fungi:bacteria
GP/GN: Gram-positive:Gram-negative
MB:TOC: microbial biomass:total organic carbon
MB:TN: microbial biomass:total nitrogen
Gn: Gram-negative bacteria
Ab: Actinobacteria
Gp: Gram-positive bacteria
Sf: saprotrophic fungi
Ef: ectomycorrhizal fungi
Mb: methanotrophic bacteria
An: anaerobic bacteria
Am: arbuscular mycorrhizal fungi.

## References

[B1] Bligh E. G. Dyer W. J. (1959). A rapid method of total lipid extraction and purification. *Can. J. Physiol. Pharm.* 37 911–918. 10.1139/y59-099 13671378

[B2] Butnan S. Deenik J. L. Toomsan B. Vityakon P. (2017). Biochar properties affecting carbon stability in soils contrasting in texture and mineralogy. *Agric. Nat. Resour.* 51 492–498. 10.1016/j.anres.2018.03.002

[B3] Che R. Wang Y. Li K. Xu Z. Hu J. Wang F. (2019). Degraded patch formation significantly changed microbial community composition in alpine meadow soils. *Soil Till. Res.* 195:104426. 10.1016/j.still.2019.104426

[B4] Chen H. Ma J. Wei J. Gong X. Yu X. Guo H. (2018). Biochar increases plant growth and alters microbial communities *via* regulating the moisture and temperature of green roof substrates. *Sci. Total Environ.* 635 333–342. 10.1016/j.scitotenv.2018.04.127 29669299

[B5] Chen J. Xuan J. Du C. Xie J. (1997). Effect of potassium nutrition of rice on rhizosphere redox status. *Plant Soil* 188 131–137. 10.1023/A:1004264411323

[B6] DeForest J. L. Snell R. S. (2020). Tree growth response to shifting soil nutrient economy depends on mycorrhizal associations. *New Phytol.* 225 2557–2566. 10.1111/nph.16299 31677163

[B7] Djukic I. Zehetner F. Mentler A. Gerzabek M. H. (2010). Microbial community composition and activity in different Alpine vegetation zones. *Soil Biol. Biochem.* 42 155–161. 10.1016/j.soilbio.2009.10.006

[B8] Dong S. K. Wen L. Li Y. Y. Wang X. X. Zhu L. Li X. Y. (2012). Soil-quality effects of grassland degradation and restoration on the qinghai-tibetan plateau. *Soil Sci. Soc. Am. J.* 76 2256–2264. 10.2136/sssaj2012.0092

[B9] El-Naggar A. Lee S. S. Awad Y. M. Yang X. Ryu C. Rizwan M. (2018). Influence of soil properties and feedstocks on biochar potential for carbon mineralization and improvement of infertile soils. *Geoderma* 332 100–108. 10.1016/j.geoderma.2018.06.017

[B10] Evgrafova S. Y. Santruckova H. Shibistova O. B. Elhottova D. Cerna B. Zrazhevskaya G. K. (2008). Phospholipid fatty acid composition of microorganisms in pine forest soils of Central Siberia. *Biol. Bull.* 35 452–458. 10.1134/s1062359008050038 18956729

[B11] Fan Q. Cui L. Quan G. Wang S. Sun J. Han X. (2018). Effects of wet oxidation process on biochar surface in acid and alkaline soil environments. *Materials* 11:2362. 10.3390/ma11122362 30477163 PMC6317012

[B12] Fang Z. Li D. D. Jiao F. Yao J. Du H. T. (2019). The latitudinal patterns of leaf and soil C:N:P stoichiometry in the loess plateau of China. *Front. Plant Sci.* 10:85. 10.3389/fpls.2019.00085 30949183 PMC6436477

[B13] Farrell M. Kuhn T. K. Macdonald L. M. Maddern T. M. Murphy D. V. Hall P. A. (2013). Microbial utilisation of biochar-derived carbon. *Sci. Total Environ.* 465 288–297. 10.1016/j.scitotenv.2013.03.090 23623696

[B14] Feng R. Long R. Shang Z. Ma Y. Dong S. Wang Y. (2009). Establishment of Elymus natans improves soil quality of a heavily degraded alpine meadow in Qinghai-Tibetan Plateau, China. *Plant Soil* 327 403–411. 10.1007/s11104-009-0065-3

[B15] Frostegard A. Baath E. (1996). The use of phospholipid fatty acid analysis to estimate bacterial and fungal biomass in soil. *Biol. Fert. Soils* 22 59–65. 10.1007/BF00384433

[B16] Fu G. Shen Z. X. (2016). Response of Alpine plants to nitrogen addition on the tibetan plateau: a meta-analysis. *J. Plant Growth Regul.* 35 974–979. 10.1007/s00344-016-9595-0

[B17] Fu G. Shen Z. X. (2017). Response of Alpine soils to nitrogen addition on the tibetan plateau: a meta-analysis. *Appl. Soil Ecol.* 114 99–104. 10.1016/j.apsoil.2017.03.008

[B18] Gul S. Whalen J. K. (2016). Biochemical cycling of nitrogen and phosphorus in biochar-amended soils. *Soil Biol. Biochem.* 103 1–15. 10.1016/j.soilbio.2016.08.001

[B19] Han F. Ren L. Zhang X. C. (2016). Effect of biochar on the soil nutrients about different grasslands in the Loess Plateau. *Catena* 137 554–562. 10.1016/j.catena.2015.11.002

[B20] Jenkins J. R. Viger M. Arnold E. C. Harris Z. M. Ventura M. Miglietta F. (2017). Biochar alters the soil microbiome and soil function: results of next-generation amplicon sequencing across Europe. *GCB Bioenergy* 9 591–612. 10.1111/gcbb.12371

[B21] Jiang X. Tan X. Cheng J. Haddix M. L. Cotrufo M. F. (2019). Interactions between aged biochar, fresh low molecular weight carbon and soil organic carbon after 3.5 years soil-biochar incubations. *Geoderma* 333 99–107. 10.1016/j.geoderma.2018.07.016

[B22] Li J. Shao X. Huang D. Shang J. Liu K. Zhang Q. (2020). The addition of organic carbon and nitrogen accelerates the restoration of soil system of degraded alpine grassland in Qinghai-Tibet Plateau. *Ecol. Eng.* 158:106084. 10.1016/j.ecoleng.2020.106084

[B23] Li S. Wang S. Shangguan Z. (2019). Combined biochar and nitrogen fertilization at appropriate rates could balance the leaching and availability of soil inorganic nitrogen. *Agric. Ecosyst. Environ.* 276 21–30. 10.1016/j.agee.2019.02.013

[B24] Li Y. Y. Dong S. K. Wen L. Wang X. X. Wu Y. (2014). Soil carbon and nitrogen pools and their relationship to plant and soil dynamics of degraded and artificially restored grasslands of the Qinghai–Tibetan Plateau. *Geoderma* 213 178–184. 10.1007/s11368-018-2192-z

[B25] Li Y. Pan F. Yao H. (2018). Response of symbiotic and asymbiotic nitrogen-fixing microorganisms to nitrogen fertilizer application. *J. Soil Sediment* 19 1948–1958. 10.1016/j.geoderma.2013.08.022

[B26] Liu S. Zamanian K. Schleuss P.-M. Zarebanadkouki M. Kuzyakov Y. (2018). Degradation of Tibetan grasslands: consequences for carbon and nutrient cycles. *Agric. Ecosyst. Environ.* 252 93–104. 10.1016/j.agee.2017.10.011

[B27] Liu X. Wang Z. Zheng K. Han C. Li L. Sheng H. (2020). Changes in soil carbon and nitrogen stocks following degradation of alpine grasslands on the Qinghai-Tibetan Plateau: a meta-analysis. *Land Degrad. Dev.* 32 1262–1273. 10.1002/ldr.3796

[B28] Lu S. G. Sun F. F. Zong Y. T. (2014). Effect of rice husk biochar and coal fly ash on some physical properties of expansive clayey soil (Vertisol). *Catena* 114 37–44. 10.1016/j.catena.2013.10.014

[B29] Luo Y. Durenkamp M. De Nobili M. Lin Q. Brookes P. C. (2011). Short term soil priming effects and the mineralisation of biochar following its incorporation to soils of different pH. *Soil Biol. Biochem.* 43 2304–2314. 10.1016/j.soilbio.2011.07.020

[B30] Ma W. Li J. Gao Y. Xing F. Sun S. Zhang T. (2020). Responses of soil extracellular enzyme activities and microbial community properties to interaction between nitrogen addition and increased precipitation in a semi-arid grassland ecosystem. *Sci. Total Environ.* 703 134691. 10.1016/j.scitotenv.2019.134691 31731161

[B31] Muhammad N. Hussain M. Ullah W. Khan T. A. Ali S. Akbar A. (2018). Biochar for sustainable soil and environment: a comprehensive review. *Arab. J. Geosci.* 11:731. 10.1007/s12517-018-4074-5

[B32] Oliveira F. R. Patel A. K. Jaisi D. P. Adhikari S. Lu H. Khanal S. K. (2017). Environmental application of biochar: current status and perspectives. *Bioresour. Technol.* 246 110–122. 10.1016/j.biortech.2017.08.122 28863990

[B33] Oram N. J. van de Voorde T. F. J. Ouwehand G.-J. Bezemer T. M. Mommer L. Jeffery S. (2014). Soil amendment with biochar increases the competitive ability of legumes *via* increased potassium availability. *Agric. Ecosyst. Environ.* 191 92–98. 10.1016/j.agee.2014.03.031

[B34] Peng F. Xue X. You Q. Huang C. Dong S. Liao J. (2018). Changes of soil properties regulate the soil organic carbon loss with grassland degradation on the Qinghai-Tibet Plateau. *Ecol. Indic.* 93 572–580. 10.1016/j.ecolind.2018.05.047

[B35] Piao S. Tan K. Nan H. Ciais P. Fang J. Wang T. (2012). Impacts of climate and CO2 changes on the vegetation growth and carbon balance of Qinghai–Tibetan grasslands over the past five decades. *Global. Planet. Change* 98-99 73–80. 10.1016/j.gloplacha.2012.08.009

[B36] Pietikainen J. Kiikkila O. Fritze H. (2000). Charcoal as a habitat for microbes and its effect on the microbial community of the underlying humus. *Oikos* 89 231–242. 10.1034/j.1600-0706.2000.890203.x 11841302

[B37] Quilliam R. S. DeLuca T. H. Jones D. L. (2012). Biochar application reduces nodulation but increases nitrogenase activity in clover. *Plant Soil* 366 83–92. 10.1007/s11104-012-1411-4

[B38] Reed E. Y. Chadwick D. R. Hill P. W. Jones D. L. (2017). Critical comparison of the impact of biochar and wood ash on soil organic matter cycling and grassland productivity. *Soil Biol. Biochem.* 110 134–142. 10.1016/j.soilbio.2017.03.012

[B39] Sadaf J. Shah G. A. Shahzad K. Ali N. Shahid M. Ali S. (2017). Improvements in wheat productivity and soil quality can accomplish by co-application of biochars and chemical fertilizers. *Sci. Total Environ.* 607-608 715–724. 10.1016/j.scitotenv.2017.06.178 28711001

[B40] Smith A. P. Marin-Spiotta E. Balser T. (2015). Successional and seasonal variations in soil and litter microbial community structure and function during tropical postagricultural forest regeneration: a multiyear study. *Glob. Chang Biol.* 21 3532–3547. 10.1111/gcb.12947 25873563

[B41] Somleva M. Tomaszewski Z. Conger B. (2002). Agrobacterium-mediated genetic transformation of switchgrass. *Crop Sci.* 42 2080–2087. 10.2135/cropsci2002.2080

[B42] Sun J. Hou G. Liu M. Fu G. Zhan T. Zhou H. (2019). Effects of climatic and grazing changes on desertification of alpine grasslands. *Northern Tibet. Ecol. Indic.* 107:105647. 10.1016/j.ecolind.2019.105647

[B43] Sun L. Li J. Wang Q. Zhang Y. Xu Z. Wang R. (2020). The effects of eight years of conservation tillage on the soil physicochemical properties and bacterial communities in a rain-fed agroecosystem of the loess plateau, China. *Land Degrad. Dev.* 31 2475–2489. 10.1002/ldr.3619

[B44] Sundh I. Borjesson G. Tunlid A. (2000). Methane oxidation and phospholipid fatty acid composition in a podzolic soil profile. *Soil Biol. Biochem.* 32 1025–1028. 10.1016/S0038-0717(99)00220-5

[B45] Wahi R. Zuhaidi N. F. Q. A. Yusof Y. Jamel J. Kanakaraju D. Ngaini Z. (2017). Chemically treated microwave-derived biochar: an overview. *Biomass Bioenerg* 107 411–421. 10.1016/j.biombioe.2017.08.007

[B46] Wang D. Huang X. Qiao N. Geng Q. Liu Y. Song H. (2020). Effects of mowing and fertilization on soil quality in a semiarid grassland of North China. *Land Degrad. Dev.* 32 1656–1666. 10.1002/ldr.3783

[B47] Wang X. G. Lü X. T. Zhang H. Y. Dijkstra F. A. Jiang Y. G. Wang X. B. (2020). Changes in soil C:N:P stoichiometry along an aridity gradient in drylands of northern China. *Geoderma* 361:114087. 10.1016/j.geoderma.2019.114087

[B48] White D. Davis W. Nickels J. King J. Bobbie R. (1979). Determination of the sedimentary microbial biomass by extractible lipid phosphate. *Oecologia* 40 51–62. 10.1007/BF00388810 28309603

[B49] Woolf D. Amonette J. E. Street-Perrott F. A. Lehmann J. Joseph S. (2010). Sustainable biochar to mitigate global climate change. *Nat. commun.* 1 56. 10.1038/ncomms1053 20975722 PMC2964457

[B50] Wu Y. Xu N. Wang H. Li J. Zhong H. Dong H. (2021). Variations in the diversity of the soil microbial community and structure under various categories of degraded wetland in Sanjiang Plain, northeastern China. *Land Degrad. Dev.* 32 2143–2156. 10.1002/ldr.3872

[B51] Yagüe M. R. Domingo-Olivé F. Bosch-Serra ÀD. Poch R. M. Boixadera J. (2016). Dairy cattle manure effects on soil quality: porosity, earthworms, aggregates and soil organic carbon fractions. *Land Degrad. Dev.* 27 1753–1762. 10.1002/ldr.2477

[B52] Yao Z. Zhao C. Yang K. Liu W. Li Y. You J. (2016). Alpine grassland degradation in the Qilian Mountains, China — a case study in damaying grassland. *Catena* 137 494–500. 10.1016/j.catena.2015.09.021

[B53] Ye Z. Liu L. Tan Z. Zhang L. Huang Q. (2020). Effects of pyrolysis conditions on migration and distribution of biochar nitrogen in the soil-plant-atmosphere system. *Sci. Total Environ.* 723:138006. 10.1016/j.scitotenv.2020.138006 32222503

[B54] Yu L. Yu M. Lu X. Tang C. Liu X. Brookes P. C. (2018). Combined application of biochar and nitrogen fertilizer benefits nitrogen retention in the rhizosphere of soybean by increasing microbial biomass but not altering microbial community structure. *Sci. Total Environ.* 640-641 1221–1230. 10.1016/j.scitotenv.2018.06.018 30021287

[B55] Zhang L. Jing Y. Xiang Y. Zhang R. Lu H. (2018). Responses of soil microbial community structure changes and activities to biochar addition: a meta-analysis. *Sci. Total Environ.* 643 926–935. 10.1016/j.scitotenv.2018.06.231 29960229

[B56] Zhang N. Zhong B. Zhao C. Wang E. Wang Y. Chen D. (2020). Change of soil physicochemical properties, bacterial community and aggregation during desertification of grasslands in the Tibetan Plateau. *Eur. J. Soil Sci.* 72 274–288. 10.1111/ejss.12939

[B57] Zhao R. Feng J. Liu J. Fu W. Li X. Li B. (2019). Deciphering of microbial community and antibiotic resistance genes in activated sludge reactors under high selective pressure of different antibiotics. *Water Res.* 151 388–402. 10.1016/j.watres.2018.12.034 30616051

[B58] Zhao Z. B. He J. Z. Geisen S. Han L. L. Wang J. T. Shen J. P. (2019). Protist communities are more sensitive to nitrogen fertilization than other microorganisms in diverse agricultural soils. *Microbiome* 7:33. 10.1186/s40168-019-0647-0 30813951 PMC6393985

[B59] Zhu C. Tian G. Luo G. Kong Y. Guo J. Wang M. (2018). N-fertilizer-driven association between the arbuscular mycorrhizal fungal community and diazotrophic community impacts wheat yield. *Agric. Ecosyst. Environ.* 254 191–201. 10.1016/j.agee.2017.11.029

[B60] Zhu L. X. Xiao Q. Shen Y. F. Li S. Q. (2017). Microbial functional diversity responses to 2 years since biochar application in silt-loam soils on the Loess Plateau. *Ecotox. Environ. Saf.* 144 578–584. 10.1016/j.ecoenv.2017.06.075 28688360

[B61] Zong N. Fu G. (2021). Variations in species and function diversity of soil fungal community along a desertification gradient in an alpine steppe. *Ecol. Indic.* 131:108197. 10.1016/j.ecolind.2021.108197

